# Global Alignment of Pairwise Protein Interaction Networks for Maximal Common Conserved Patterns

**DOI:** 10.1155/2013/670623

**Published:** 2013-03-27

**Authors:** Wenhong Tian, Nagiza F. Samatova

**Affiliations:** ^1^School of Computer Science and Engineering, University of Electronic Science and Technology of China, Chengdu 611731, China; ^2^Department of Computer and Mathematics Division, Oak Ridge National Laboratory, Oak Ridge, TN 37831, USA; ^3^Computer Science Department, North Carolina State University, Raleigh, NC 27696, USA

## Abstract

A number of tools for the alignment of protein-protein interaction (PPI) networks have laid the foundation for PPI network analysis. Most of alignment tools focus on finding conserved interaction regions across the PPI networks through either local or global mapping of similar sequences. Researchers are still trying to improve the speed, scalability, and accuracy of network alignment. In view of this, we introduce a connected-components based fast algorithm, HopeMap, for network alignment. Observing that the size of true orthologs across species is small comparing to the total number of proteins in all species, we take a different approach based on a precompiled list of homologs identified by KO terms. Applying this approach to *S. cerevisiae* (yeast) and *D. melanogaster* (fly), *E. coli* K12 and *S. typhimurium*, *E. coli* K12 and *C. crescenttus*, we analyze all clusters identified in the alignment. The results are evaluated through up-to-date known gene annotations, gene ontology (GO), and KEGG ortholog groups (KO). Comparing to existing tools, our approach is fast with linear computational cost, highly accurate in terms of KO and GO terms specificity and sensitivity, and can be extended to multiple alignments easily.

## 1. Introduction

**Figure 1:**
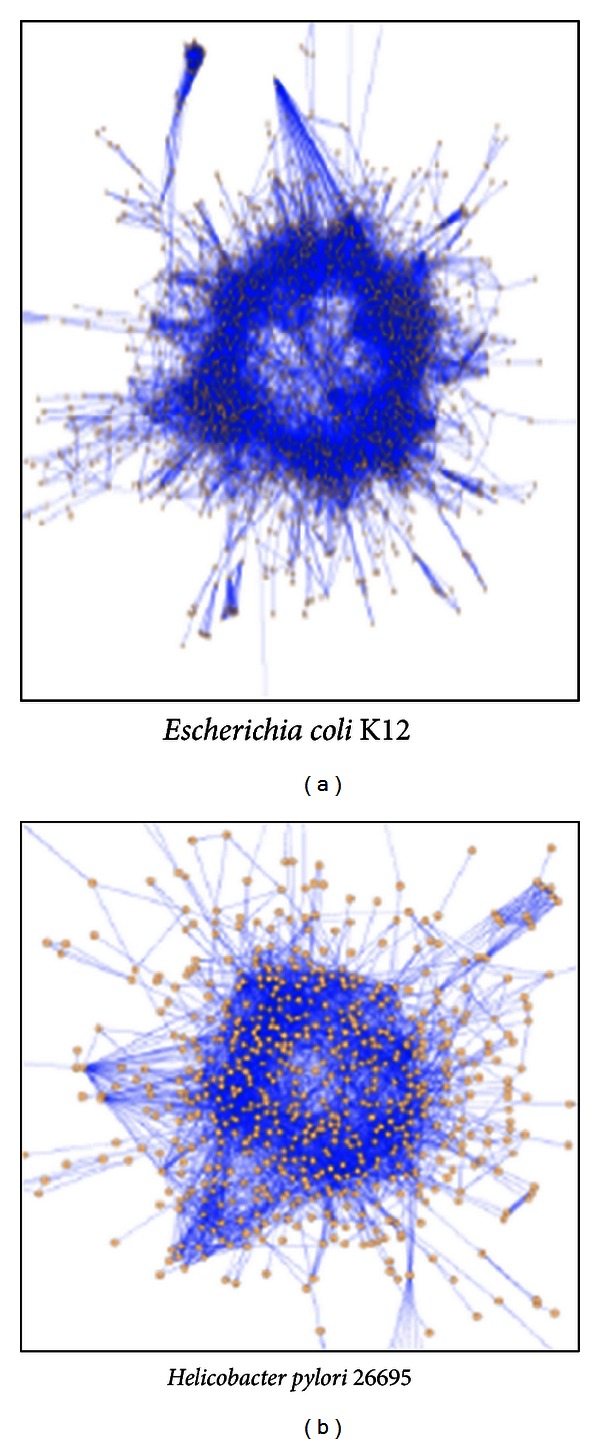
global visualization of protein interaction networks (from [15]).

Protein-protein interactions (PPI) are of central importance for virtually every process in a living cell. For example, information about these interactions improves our understanding of diseases and can provide the basis for new therapeutic approaches [1]. One of fundamental goals of system biology is to understand how proteins in the cell interact with each other. However, finding all protein interactions is costly and labor intensive. For example, to find all pairwise interactions for a species with 5000 proteins, one needs to do 12497500 pairwise tests. This is one reason that current known direct interactions are incomplete. High-throughput experimental techniques (e.g., yeast two-hybrid and coimmunoprecipitation test) can be helpful in this case. Integrated probability models are also used to predict the protein-protein interactions [1, 2]. Quite a few databases, DIP [3], IntAct [4], BioGRID [5], HPRD [6], and IntPro [7], are public available for collecting and storing PPI network data. Researchers [1, 8–14] are trying to identify conserved patterns such as ortholog groups and functional similar pathways/complexes across species using PPI network data.  Figure 1 provides an example of global visualization of protein interaction networks.

The exact solution of identifying conserved regions across species, that is, the network alignment problem, is NP-hard [1, 8–14]. This challenge attracts many researchers to find efficient heuristic solutions for the problem.

A powerful way of representing and analyzing all PPI network data is to use network models and classical graph-theoretical approaches [16, 17]. In a PPI network, each protein is represented as a node and a direct physical interaction between proteins by an edge. When identifying conserved patterns across PPI networks, highly similar sequence proteins (homologues) are firstly identified, then conserved interactions are clustered, and finally functional similarities of each cluster should be validated.

An interesting hypothesis is that highly similar sequence proteins (homologues) within a species and across species may perform similar functions. This needs to be justified by experimental, comparative, and statistical study. This is one of major purposes of our research. Statistical and computational analyses of these networks by combination with gene expression data can be used for inferring biological function categories, gene ontology, orthologs, clusters, and so forth [1, 8–14, 18].

Statistical and comparative analysis of PPI networks across species is proved to be a valuable tool [1, 8–14]. Such analysis provides more information and easier tools beyond traditional sequence-based comparative genomic analyses. Such an analysis can identify conserved interaction regions, predict protein interactions and functions, and provide gene ontology (GO) enrichment and so forth.

Most of previous network alignment tools focus on conserved functional patterns cross species [1, 8, 11, 12] or on maximizing the overall match between PPI networks globally such as Isorank [13] and Isorank-M [14]. The existing tools firstly find the conserved interaction regions by search algorithms and then validate the results by gene annotations such as gene ontology (GO) [19] or KEGG ortholog (KO) groups [20]. We observe that results that are good for one annotation (KO, e.g.) may not be good for another (GO). Also scoring functions that work for one set of parameters of an alignment may not work for others [8, 9], and most often manual tuning is needed. Researchers are still trying to improve the speed and accuracy of network alignment. In view of this, we introduce an iterative connected-components based algorithm, HopeMap, aiming to improve the speed, scalability, and accuracy of the alignment and also propose a generic scoring system.

In this paper, we explore a new approach to comparative analysis of PPI networks with focus on clustering and validating functional homologues (genome) with conserved interactions (interactome). We firstly consider the network alignment to globally identify conserved interaction in the close homologues. By close homologues, we mean proteins in the same homologs are highly similar in terms of sequence similarity, for example, BLAST *E* value is much smaller than *e* ^−7^. To this end, KO groups [20] (and INPARANOID [21] for pairwise) are used for clustering ortholog groups across species. Even if protein-protein interaction databases continue to grow in size and species coverage, the size of true homologous groups will not increase too much but stay manageable. For example, the current common number of KO terms for Baker's yeast (4738 proteins in PPI network) and fruit fly (7165 proteins in PPI network) is about 767. KO group is a dynamic system that can easily accept new genes once they are identified.

**Figure 2:**
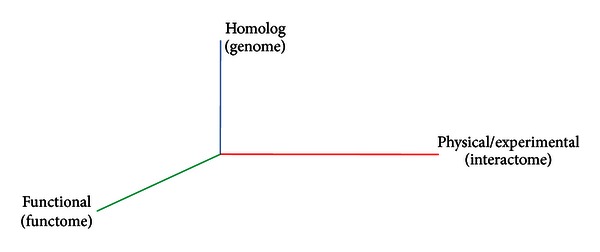
3D alignment guided by physical interaction (interactome), sequence similarity (genome), and functional coherence (functome) of proteins in the network.

As shown in Figure 2, we propose a graph-based algorithm for the network alignment. This clustering and alignment algorithm combines information of homolog (genomic similarity), physical interactions conservation, and equivalent functions (HOPE), and we call our main algorithm HopeMap.

**Pseudocode of the connected-component algorithm.**
(1) Input: Graph *G* = (*V*, *E*), Start node *v0 *
(2) index = 0     // DFS node number counter
(3)  *S* = empty    // An empty stack of nodes
(4)   tarjan (*v0*)   // Start a DFS at the start node
(5)   procedure tarjan (*v*)
(6)  *v*.index = index  // Set the depth index for *v *
(7)  *v*.lowlink = index
(8)  index = index + 1
(9)  *S*.push (*v*)    // Push *v* on the stack
(10)  For all (*v*, *v*′) in *E* do // Consider successors of *v *
(11)   if (*v*′.index is undefined) // Was successor *v*′ visited?
(12)   tarjan (*v*′)   // Recurse
(13)   *v*.lowlink = min (*v*.lowlink, *v*′.lowlink)
(14)  elseif (*v*′ in *S*)     // Is *v*′ on the stack?
(15)   *v*.lowlink = min (*v*.lowlink, *v*′.index)
(16)  if (*v*.lowlink == *v*.index) // Is *v* the root of an SCC?
(17)  print “SCC:”
(18)  repeat
(19)   *v*′ = *S*.pop
(20)   print *v*′
(21)  until (*v*′ == *v*)

### 1.1. Contributions

Our contributions may be in threefolds: (1) developing a fast, accurate, and general tool for the network alignment, which can work for both pairwise and multiple species. Fast means linear computation cost; accurate means the aligned results are highly correct in terms of known functional annotations; general means the clustering algorithm is parameter-free and can work for other networks as well. (2) Proposing a generic scoring system, an open system to accept new features and to refine the identified conserved regions across species. (3) Introducing a multiple-validating approach for functional homologues using known annotated orthologs and pathways/complexes.

Our approach and focus are different from existing tools. The existing tools firstly find the conserved interaction regions by search algorithms and then validate the results by gene annotations (GO, KO). Observing that the size of true orthologs cross species is small comparing to the total number of proteins in all species, we take a different approach in three steps: starting from the similar orthologous clusters across species, finding conserved interaction regions iteratively with a generic scoring system, and validating results across multiple known functional annotations. Using known ortholog clustering results such as KO groups reduces the computational cost of finding sequence similar proteins. Applying connected-components based algorithm to find conserved region across species assures a fast approach to maximize matches across species; it is also a parameter-free clustering algorithm, unlike most of existing tools, which need setting different sets of parameters for different alignment. Iterative process can be applied to refine the identified regions. The generic scoring function is an open system; currently it combines evolutionary evidence such as genomic, interaction, and functional similarities. It can incorporate more features in the future if available and necessary.

### 1.2. Related Work from Pan-Similar Sequences to Functional Similar Homologues

In general, network alignment may be classified using following criterions:

1. in terms of the number of networks aligned: pairwise or multiple;
2. in terms of number of nodes aligned simultaneously: local or global;
3. in terms of guided models: divergence/duplication evolution, neighbouring topology in the PPI networks, and functional categories MIPS (or GO, KO, orthologs, etc.).

Table 1 shows a summary of classification of different network alignment tools. Some tools use the combination of previous three criterions. Our tool currently focuses on global pairwise alignment guided by interacting and functional similar homologues. Extending our tool to multiple alignments is easy. The history and some future directions in the network alignment were reviewed in [2].

Alignment of protein-protein interaction networks passed through two generations: in the first generation, conserved pathways/complexes between two species are indentified, often called pairwise alignment. PathBLAST [11] is one of the pioneering works in this field and one of the first generation tools in this line. NetworkBLAST [2] extends PathBLAST to align up to three networks and introduces probability model for interactions. MaWish focuses on divergence/duplication model guided by the evolution. Graemlin 1.0 introduces an integration probability model to predict the interactions and can align more than three networks. Above-mentioned tools are also called local alignment because their search algorithms for the conserved regions start from small local regions and then greedily expand. Later, Isorank introduces the global alignment concept by adopting Google Pagerank algorithm idea to the network alignment. Comparing to global alignment that finds maximum matches across species, local alignment may just find maximal results. Another key issue for local search algorithms (NetworkBLAST, NetworkBLAST-M [10], Graemlin 1.0, and Mawish) is that they produce many overlapped subnetworks which need to be filtered. The paper [22] applies graph kernels for pairwise alignment, and [23] extends Mawish pairwise alignment.

The first generation aligning tools focus on identifying conserved interaction regions across species. However, the accuracy, scalability, and scoring functions of the first generation tools still have limitations. Most of aligned results do not have high sensitivity or specificity in terms of biological relevance. The first generation tools also used very crude measurement for the specificity and sensitivity; for example, a cluster that has more than three proteins with same complexes IDs in MIPS (NetworkBLAST) or KEGG (Graemlin 1.0) is called a pure cluster or correct equivalence class; and if more than half proteins in a cluster have same GO terms, the cluster is called GO enrichment.

The second generation aligning tools, such as Graemlin 2.0 and NetworkBLAST-M, are trying to improve both accuracy and speed. Previous tools other than NetworkBLAST-M are also called progressive alignment approach with exponential possible representations of every set of potential orthologous proteins, which make them slow and inefficient of using memory. The computational cost of major algorithm in Graemlin 2.0 is claimed to be linear with the number of proteins and PPIs in all the species. Our major algorithm is linear with the number of nodes and edges in the alignment graph, which will be introduced later.

As reported in Graemlin 2.0, which may be the only one to compare results of all tools against KO groups, the specificity of NetworkBLAST, Graemlin 1.0, MaWish, Isorank, and Graemlin 2.0 is varying from species to species with average accuracy (42%, 53%, 57%, 70%, and 81%). However, only KO (Graemlin 2.0) or GO terms (NetworkBLAST-M) are used in each tool. It is known that a tool that works fine for one term (KO or GO) may be not good for another term (MIPS FunCats [24], e.g.). We introduce multiple validation through more than one functional annotation; that is, we align networks using KO groups and validate through GO terms and MIPS [25] complexes.

Most of existing tools use different scoring systems to sort the identified subnetworks. NetworkBLAST and Graemlin 1.0 combine testing and prediction of interaction probabilities in their scoring functions. Isorank uses network structure and sequence similarity information to score each node. Notice most of scoring systems need manual parameters tuning, and Graemlin 2.0 develops an automatic parameters learning scoring system. We propose a generic scoring function. Currently it combines evolutionary evidence such as genomic, interaction, and functional similarities. It can incorporate more features in the future if available and necessary.

## 2. Network Alignment Problem Formulation

The network alignment problem has been formulated formally. While there are some variations from one tool to another, the major ideas are similar: combing gene-sequence information and PPI network information to find conserved interaction regions across species. Generally speaking, firstly similar sequences proteins (based on BLAST scores) are put into equivalence class groups which may have one-to-many proteins from a single species, then alignment tools are used to identify conserved interaction regions across species, and finally KO groups or GO terms are used to validate the results.

Each PPI network may be represented as an undirected graph *G* = (*V*, *E*) where *V* is the set of nodes and *E* is the set of edges. (*G* may be a weighted graph; i.e., a weight measure *w*(*e*) may be associated with each edge *e* in *E*). The biological interpretation of network alignment is to find functional orthologs or homologues across different organisms. In analogy with sequence alignment, local and global network alignment is defined in [9, 13, 14]. In this paper, the global network alignment approach is applied.

Network comparative study or alignment helps to interpret cellular machineries such as identifying common conserved interaction pathways or complexes across two or more species. Although the general problem is NP-hard, heuristic methods with the combination of sequence, interaction, and functional similarities can be developed to tackle it. *A network alignment graph* can be built across two or more species based on protein sequence similarity, interaction conservation, and functional coherence. The nodes in the *alignment* graph represent sets of proteins, ideally one from each species and edges for the conserved PPIs across the compared species. The heart of network alignment algorithms is to find the highly conserved interaction regions across homologs among different species.

**Figure 3:**
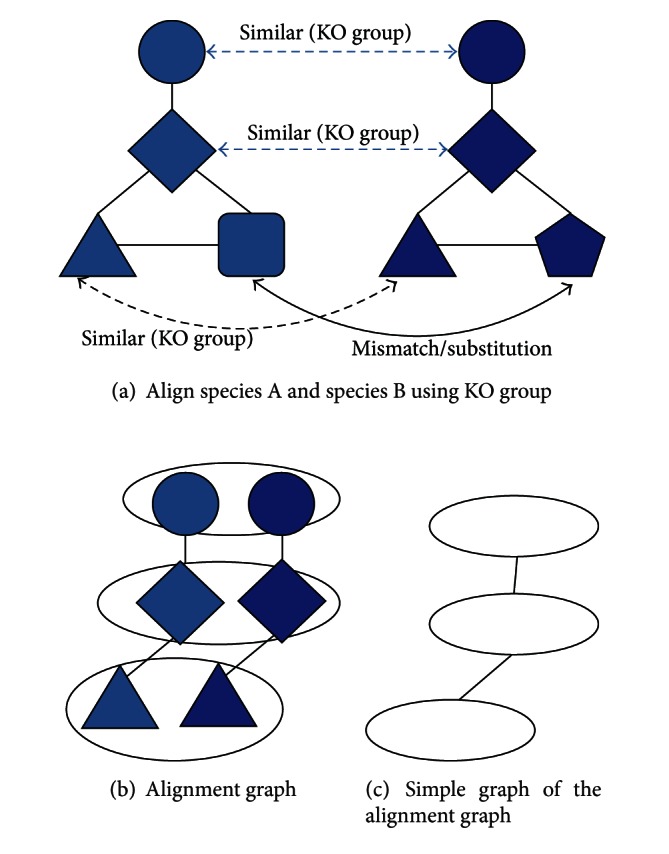
Building an alignment graph.

As shown in Figure 3, using KO group for similarity, three nodes alignment graph of species A and B can be built, and each node in the alignment graph has two proteins in this example. Then alignment graph can be simplified as a normal graph, and connected-components based graph algorithm therefore can be applied. Connected components in this case represent conserved patterns. Once connected components are found in the simple graph, we can go back to alignment graph and original protein interaction network of each species to identify conserved patterns in each species.

Highly similar sequence proteins are believed to perform same biological functions across species. We use them as the starting point of alignment to find interactions conserved between orthologs across species. The results are more biological relevant and can be evaluated through known functional categories or gene ontology.

**Figure 4:**
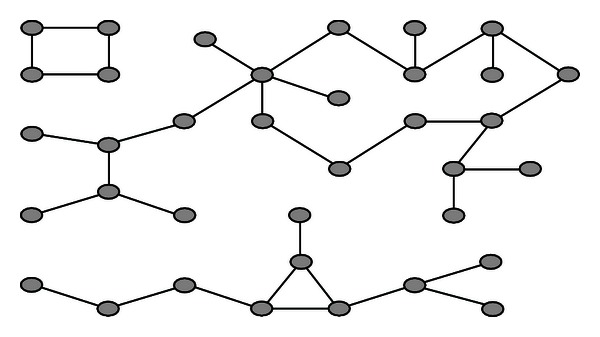
Three connected components in a graph.

In graph theory, a *connected component* of an undirected graph is a subgraph in which any two vertices are connected to each other by paths, and which is connected to no additional vertices. For example, the graph shown in Figure 4 has three connected components. Especially a graph that is itself connected has exactly one connected component, consisting of the whole graph.

It is straightforward to compute the connected components of a graph in linear time using either breadth-first search or depth-first search. To find all the connected components of a graph, loop through its vertices, starting a new breadth first or depth first search whenever the loop reaches a vertex that has not already been included in a previously found connected component.

## 3. Our Algorithm: HopeMap

**Figure 5:**
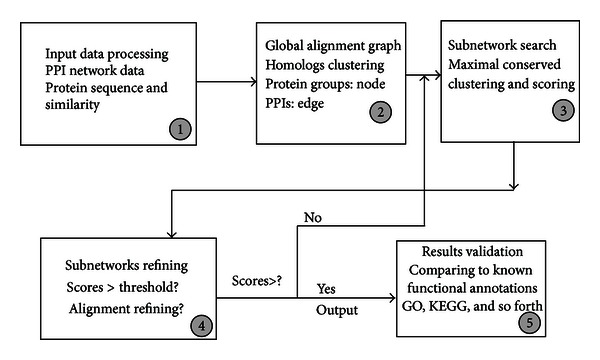
HopeMap network alignment in five steps.

Our algorithm, called HopeMap, can be described as follows (Figure 5 shows the five-step flow of HopeMap).

(1) *The First Step.* Obtaining the PPI networks data and preprocessing them, DIP, IntAct, and SNDB [26], and UniProt [27], are some of PPI network databases. We need to find all protein pairs that are interacting with each other in a species and store them in a sparse matrix to save space. All-against-all BLAST scores for all the species can be obtained for homolog clustering of the next step.

(2) *The Second Step.* Finding highly similar sequences proteins across species. Using homolog clustering to identify homolog groups across different species based on all-virus-all BLAST scores and ortholog annotations. To this end, existing tools such as KO groups and INPARANOID can be used. Our approach can use any reliable ortholog annotations available; it is an open system. KO groups are one of the best-known functional ortholog group annotations across species. Besides sequence similarity (homolog), all the genes in the same KO groups perform same functions. Once homolog groups are identified across species, a network alignment graph can be built based on them. The nodes in the graph represent sets of proteins, ideally one from each species in the same homolog group and edges for the conserved PPIs across the compared species. One way of adding edges between node pairs (*a*_1_, *a*_2_) and (*a*_2_, *b*_2_) is when both (*a*_1_, *b*_1_) and (*a*_2_, *b*_2_) are directly interacting with each other in their PPI networks. Other rules for adding edges can be incorporated such as those introduced in NetworkBLAST.

(3) *The Third Step.* Identifying conserved protein interaction regions in the alignment graph. Global alignment of proteins homolog groups is applied to identify conserved interaction regions. The major algorithm is based on finding connected components (clusters) in the alignment graph. The basic idea of the connected-component algorithm (Pseudocode 1) is a depth-first search begins from a start node. The strongly connected components form the subtrees of the search tree, the roots of which are the roots of the strongly connected components. The nodes are placed on a stack in the order in which they are visited. When the search returns from a subtree, the nodes are taken from the stack, and it is determined whether each node is the root of a strongly connected component. If a node is the root of a strongly connected component, then it and all of the nodes are taken off before it forms that strongly connected component.

(4) *The Fourth Step*. Once connected components (clusters) in the alignment graph are identified, our scoring system is used to find high scores subnetworks (clusters). Our scoring functions of clusters combine genomic similarity score, interaction conservation, and functional coherence. It is a normalized function with values in interval [0, 1], so that it is convenient to compare the scores of different clusters. The scoring function of a cluster *C* is defined as

(1) $$\begin{array}{c} \text{Score}\left(C\right)\;=\;w_{1}S\left(C\right)+w_{2}I\left(C\right)+w_{3}F\left(C\right), \end{array}$$

where *S*(*C*) is the sequence similarity score or the average confidence of homolog nodes in cluster *C*, *I*(*C*) is the interaction conservation coefficient of cluster *C*, *F*(*C*) is the functional coherence score of cluster *C*, (*w*_1_, *w*_2_, *w*_3_) is the corresponding weight coefficient of *S*(*C*),  *I*(*C*), and *F*(*C*), and one-third for each is set as the default.

*Similarity Scoring S*(*C*)*: Node Scoring.* We find highly similar proteins cross species by identifying close homologues first. For each node (one protein from each species) of cluster *C* in the alignment graph, we can find their confidence score based on BLAST scores or ortholog confidence score as follows:

(2) $$\begin{array}{c} S\left(C\right)=\frac{\sum_{k=1}^{k=|C|}s\left(k\right)}{\left|C\right|}, \end{array}$$

where *s*(*k*) is the similarity (or ortholog) confidence score of node *k* in cluster *C* and |*C*| is the size of cluster *C*. Normalization of confidence scores can be done as follows: the average BLAST score of each cluster is defined as the total BLAST scores of all nodes in a cluster divided by the size of the cluster. If a node's BLAST *E* value is smaller than the average BLAST score, its confidence value is set to 1; otherwise, its confidence value is set to ratio of the average BLAST score to its *E* value. Similar approach is applied to each cluster. For simplicity, the confidence score of a cluster in the alignment graph is set to 1 if all nodes of the cluster are in the same known ortholog annotation such as KO groups or INPARANOID; otherwise the confidence score is set to zero. The same idea can be extended to other ortholog terms.

*Interaction Conservation Scoring I*(*C*)*: Node and Edge Scoring.* The conserved interactions are edges connecting all nodes in an identified cluster of the alignment graph. We use only the ratio of direct interactions conserved in a local cluster; that is, if all the proteins in a cluster are directly connected, *I*(*C*) is set to 1.0; otherwise, *I*(*C*) is set to the portion of the direct interactions conserved in the clusters. *I*(*C*) is formally defined as

(3) $$\begin{array}{c} I\left(C\right)=\frac{i\left(C\right)}{\left|C\right|\left(\left|C\right|-1\right)/2}, \end{array}$$

where *i*(*C*) is the total number of conserved interactions (defined previously), that is, the total number of edges in cluster *C*, |*C*| is the number of nodes in the cluster *C*, and |*C*|(|*C*| − 1)/2 is cliqueness measurement of the cluster *C*; that is, if cluster *C* is a clique (a complete graph), then there are (|*C*|(|*C*| − 1)/2) interactions (edges) connecting all nodes.

Since we are comparing scores of all identified subnetworks, normalization (similar to the sequence similarity) may be needed; that is, we compute the interaction conservation coefficient *I*(*C*) first for all the clusters, and then we normalize the *I*(*C*) over all clusters. If the *I*(*C*) of a cluster is larger than the average *I*(*C*), the *I*(*C*) is reset as 1.0; otherwise, the *I*(*C*) is reset to the ratio of its original value to the average value of all clusters.

*Functional Coherence Scoring F*(*C*)*: Node and Edge Scoring.* Known functional annotations such as GO terms, FunCats and complexes in MIPS, pathways/complexes in KEGG can be used as functional coherence score of a cluster. Currently, we use set intersection over union of the number of GO biological process terms covered in a cluster of a local species as the *F*(*C*) as follows:

(4) $$\begin{array}{c} F\left(C\right)=\frac{\text{Intersection}\left(\text{GO}\cdot \text{process}\cdot \text{terms}\cdot \text{in}\cdot C\right)}{\text{Union}\left(\text{GO}\cdot \text{process}\cdot \text{terms}\cdot \text{in}\cdot C\right)}. \end{array}$$

The larger the *F*(*C*), the higher the significance of the cluster *C* in GO process coherence. Normalization similar to the one used in sequence similarity scores can be applied here for all compared species too.

*Statistical Significance Assessment.* To measure the statistical significance of the score functions, for each cluster (or ortholog pairs), we randomly sample *N* clusters of the same size and compute the corresponding scores. Then we find empirical *P* value of each cluster using the methods introduced in [28, 29]. Typically, the empirical *P* value can be estimated as *P* = (*R* + 1)/(*N* + 1) where *N* is the total number of random samples and *R* is the number of these samples that produce a test statistic greater than or equal to the value for the actual data. Finally the score function can be formulated as follows:

(5) Score(C)=w1RS(C)+1NS(C)+1 +w2RI(C)+1NI(C)+1+w3RF(C)+1NF(C)+1,

where *N*_*S*(*C*)_ is the number of sample clusters (ortholog pairs), *R*_*S*(*C*)_is the number of clusters which have values larger than *S*(*C*), and (*N*_*I*(*C*)_, *R*_*I*(*C*)_) and (*N*_*F*(*C*)_, *R*_*F*(*C*)_) are similarly for *I*(*C*) and *F*(*C*), respectively. For equality and simplicity, one-third is set as default for the weight coefficient of each of three functions.

*Note.* The three factors in the scoring system can be applied alone or in combination. (1)  *S*(*C*) currently measures the functional ortholog groups across species using KO groups. Basically genes in the same KO groups have same function. Using *S*(*C*) alone shows how well the nodes in the alignment graph belong to the same functional orthologs. Graemlin 2.0 [5] therefore measures their nodes in alignment graph using KO groups. (2)  *I*(*C*) is used to measure the interaction conservation across species. The higher the *I*(*C*) is, the more interaction conservation is in a cluster. (3) Using *S*(*C*) + *I*(*C*) is enough to identify the maximum (global) conserved interaction regions across species. (4)  *F*(*C*) currently measures roughly the functional coherence of a cluster in terms of GO biological processes. The higher the *F*(*C*) is, the more significant of the cluster is regarding GO terms. Using *F*(*C*) alone, we can filter some clusters with low scores (e.g., zeros) to improve the specificity of all identified clusters regarding functional coherence. (5) Iteration may be needed to improve the results based on the combination of three factors. Termination conditions (convergence conditions) can be based on the required results.

(5) *The Fifth Step.* Results validation. Since our homolog groups are based on known annotation KO groups, currently we evaluate functional coherence of the identified local clusters in each species using gene ontology (GO). This is part of local alignment and refinement. To this end, GO TermFinder tool [30] is used, which computes empirical enrichment *P* values and corrected values for multiple testing using the false discovery rate procedure. Similar to NetworkBLAST-M, to measure the specificity of the results in terms of biological process in GO, the percent of process coherent clusters in each species is computed. The number of distinct GO categories covered in all the clusters is used as the sensitivity metric. Other known functional annotations such as FunCats and complexes in MIPS, pathways/complexes in KEGG may also be used as multiple validation.

After the fourth step, local alignment in each species can be iteratively applied to improve the score of a cluster if necessary. If an identified cluster has score less than a threshold (e.g., 0.5), our HopeMap algorithm can be used iteratively to refine clusters until the score of each cluster is higher than the threshold. Or GO biological process terms covered in each cluster can be used as the indicator to keep or remove the cluster. To be more specific, we can use the intersection over union (*I*/*U*) number of GO biological process terms covered in each cluster as the threshold to keep or remove the cluster regarding GO terms. If the value of *I*/*U* is zero, we can remove the cluster, otherwise keep it. We call this process iterative connected-components finding (ICCF). Only two iterations are currently needed for the convergence.

Our major algorithm is based on strongly connected components, and it is well known that the computational cost of strongly connected-components algorithm is linear with the number of nodes and edges in the alignment graph [31, 32].

## 4. Results Analysis

### 4.1. Inputs: Data Mining from Dozens of Huge Databases

Same as the interactome data is incomplete and noisy, the information for the protein/gene sequences, similarity, functional categories, and orthology groups currently is incomplete and inconsistent from databases to databases. Even names and IDs are not used globally but locally from one database to another. It is time consuming to find useful information from one database and cross database. Postprocessing is indeed necessary. Notice that the number of protein interactions in BioGRID is much larger than those in DIP (Isorank used both). So more numbers (quantity) of interactions conservation found in one tool may not mean that the results are better (in quality) than others.

We download the PPI interaction data from DIP and the Stanford Network Database (SNDB). We ran pairwise alignments of *yeast* (*sce*) and *fly* (*dme*) DIP networks, *Escherichia coli K12* (*eco*) and *Salmonella typhimurium LT2* (*stm*) SNDB networks, and *E. coli* (*eco*) and *Caulobacter crescentus* (*ccr*) SNDB networks. We also ran a three-way alignment of the *yeast* (*sce*), *fly* (*dme*), and *worm* (*cel*) DIP networks. We used both KO groups and GO terms for our alignment comparison metrics.  Table 2 provides a summary of all PPI networks analyzed in this paper.

### 4.2. Alignment Results

In Table 3, results of 1, 117 clusters with node size larger than one, were obtained from the supplementary material of original publication [15] using networkBLAST and INPARANOID ortholog groups. The results of 2 HopeMap-bestPairs were obtained using the same node data from 117 clusters but choosing 1314 best matching ortholog pairs only; edges are added between node pairs (*yeast1*, *fly1*) and (*yeast2*, *fly2*) only when there are edges between both *yeast* pairs (*yeast1*, *yeast2*) and *fly* pairs (*fly1*, *fly2*). Results of 3 were obtained using HopeMap, the same data from 1, and similar way as NetworkBLAST to add edges in the alignment graph. The results of 4, HopeMap-KO, were obtained using KO groups as ortholog groups, and no more interactions are added in original PPI networks from DIP.

NetworkBLAST used different techniques to add edges in the alignment graph, so that its total number of conserved regions is larger than HopeMap. Notice that the number of conserved regions is just a crude indicator since it depends on the size of regions. Table 3 shows that specificity and sensitivity in GO terms of HopeMap is comparable to NetworkBLAST, while HopeMap is simpler and faster. Using KO groups in HopeMap improves the specificity.

In Table 4, we use PPI network data from SNDB for two pairs, *E. coli K12* (*eco*)/*S. typhimurium* (*stm*) and *E. coli K12* (*eco*)/*C. crescenttus* (*ccr*). Interaction probability above 0.5 is set as the cutoff. The total number of conserved regions only includes those sizes larger than two for *eco/stm,* while regions with size larger than one are included for *eco/ccr*. High specificity and sensitivity were obtained. The reason may be that PPI network data from SNDB is quite complete.

The comparison of KO groups against other tools is provided in Tables 5 and 6. In Table 5, we provide specificity comparison in terms of KO groups for different global alignment tools (comparing to other local alignment tools which are also available in Graemlin 2.0). The metrics of specificity and sensitivity in terms of KO groups are introduced in Graemlin 2.0. In short, an equivalence class is defined as correct if all protein members in it are in the same KO group. Then the fraction of equivalence classes that were correct is shown as *Ceq,* while the fraction of nodes that were in correct equivalence classes is *Cnode* in Table 5. Other results than HopeMap are from original publication of Graemlin 2.0 table where Gr2.0 is Graemlin 2.0, ISO is Isorank, and GrG is another version of Graemlin introduced in Graemlin 2.0.

In Table 6, we provide sensitivity comparison in terms of KO groups for different global alignment tools. Other results are from original publication of Graemlin 2.0 table. Same names annotations are used as Table 5. Here *Cor* standards for the total number of nodes in correct equivalence classes, and *Tot* is the total number of equivalence classes with *r* species for *r* = 2, …, *n*.

Since HopeMap uses KO groups for homologue clustering, the results have higher specificity and sensitivity than other tools.

One observation is that all these measure and calculation of enrichment are imperfect measures of specificity and sensitivity, but they work as rough guides to validate that an aligner is not sacrificing specificity to increase sensitivity or vice versa.

In Table 7, we provide alignment comparison of *yeast/fly/worm* for NetworkBLAST, NetworkBLAST-M, and HopeMap. The results of NetworkBLAST and NetworkBLAST-M are from original publication of NetworkBLAST-M. Notice that the specificity of *C. elegans* is smaller than others in all methods. One reason may be that the PPI network data is incomplete for it: only about 3393 PPIs are recorded in DIP for 3029 proteins of *C. elegans* (worm).

For adding edges in alignment graph, HopeMap uses the following rule: for any node pair, node  (*y*_1_, *f*_1_, *w*_1_), and node  (*y*_2_, *f*_2_, *w*_2_), the edge between them added at least two pairs from three species that are directly interacting, and another pair has distance at most two in its original PPI networks. NetworkBLAST and NetworkBLAST-M used different techniques to add edges in the alignment graph. Table 7 shows that in term of specificity, HopeMap is comparable to NetworkBLAST and NetworkBLAST-M (which has higher specificity and sensitivity than Graemlin 2.0 as reported in NetworkBLAST-M). HopeMap is faster than NetworkBLAST and NetworkBLAST-M.

In Table 8, we provide another functional annotation validation. Using MIPS complexes and FunCats, we compared the identified clusters of *yeast/fly* using HopeMap-bestPairs in Table 3, where all members in a cluster that are also in MIPS complexes are counted as 100% coverage; otherwise the cluster is said not to be covered in MIPS complexes. similar measure for MIPS FunCats can also be applied. Then we find the percent of total clusters identifed by HopeMap is also covered in MIPS complexes and FunCat.

Table 9 shows typical running time of HopMap pairwise alignment when using a desktop that has 2 Ghz Pentium CPU, 1 G byte memory, and 300 G byte hard disk.

## 5. Conclusion

Based on genome similarity across different species, interactome conservations, and functional coherence, we developed a pairwise network alignment tool, called HopeMap, to improve the speed, accuracy, and generality of the alignment. HopeMap is fast; it is linear in terms of the number of nodes and edges in the alignment graph. Our results show that HopeMap has specificity and sensitivity comparable with the existing best-performing tools. Especifically, in terms of GO terms' enrichment, HopeMap performs comparably with NetworkBLAST, and HopeMap has higher specificity and sensitivity in terms of KO groups' enrichment than the other tools. Our scoring system is generic, and the main algorithm is parameter-free. HopeMap is also extensible to multiple network alignment.

## Supplementary Material

1. Score[c]=1/3∗S[c]+1/3∗I[c]+1/3∗F[c] where S[c] is the average similarity scores of all nodes in a cluster using BLAST scores or ortholog groups confidence scores, I[c] is the interaction conservation coefficient measured by the total number of interactions in a cluster divided by the |c|(|c|-1)/2, F[c] currently is the interaction over union of number of GO biological process terms covered in a cluster. These are explained the main paper too.
2. 26 clusters of Yeast-Fly pair (excel sheet “ScoreFor26ClustersYeastFly” ) are obtained using Hopmap with KO groups, also sheet “STM_ECO58ClustersScores”, “ECO_CCR42 ClustersScores”.
3. 79 clusters of Yeast-Fly (excel sheet “SoreFor79Clusters”) are obtained using Hopmap and data from NetworkBLAST.
4. GO termFinder results for each pair are provided, including excel sheet “YeastFlyIn26ClustersGO0.05”, “YeastFlyIn117ClustersGO”, and “YeastFly79ClusterGO0.05”, “Graemlin1.0Sce_Dme”, “Graemlin1.0ECO_STMGOResults”, “Graemlin1.0CCR_ECOGOresults”, as their sheet names suggest.
5. P-value for S[c]=(r*+*1)/(n*+*1) where r is the number of protein pairs which are in the same KO groups, n is the total number of possible pairs.
6. P-value for I[c]=(r*+*1)/(n*+*1) where r is the number of clusters which have values greater than or equal to the value of the actual cluster, n is the total number of random sample clusters
7. P-value for F[c]=(r*+*1)/(n*+*1) where r is the number of clusters which have values greater than or equal to the value of intersection over union of the number of GO terms in the cluster, n is the total number of random sample clusters
8. In all cases, n is large enough (3000-5000) so that the result will be statistically meaningful.

## Figures and Tables

**Table 1:** A summary of classification of different network alignment tools.

Tools	Local	Global	Pairwise	Guided models
PathBLAST	×		×	Evolution
NetworkBLAST	×			Evolution
NetworkBLAST-M	×			Evolution
MaWish	×		×	Duplication/divergence
Graemlin 1.0	×			General evolution
Graemlin 2.0		×		Evolution, duplication, and so forth
Isorank		×	×	Evolution
Isorank-M		×		Evolution
HopeMap		×	×	Evolution, function

Note*. For multiple alignment of HopeMap, only results of *yeast/fly/worm* are provided in this paper. We are preparing more results in ongoing research. In HopeMap, the global alignment concept is same as in Isorank and Graemlin 2.0.

**Table 2:** The PPI networks analyzed in this paper.

Species (tax id, short name)	Number of proteins	Number of PPI	Source
*E. coli* K12 (83333, eco)	4121	216426	SNDB [22]
*S. typhimurium* (99287, stm)	4239	94609	SNDB [22]
*C. crescentus* (190650, ccr)	3365	40524	SNDB [22]
Yeast (4932, sce)	4738	15417	DIP [13]
Fly (7227, dme)	7165	23484	DIP [13]
Worm (6239, cel)	3029	3393	DIP [13]

**Table 3:** A comparison of NetworkBLAST and HopeMap for yeast/fly.

Methods	Specificity (%) in GO	Specificity (%) in KO	Specificity (%) in INPARANOID	Number of GO categories enriched	Total conserved regions	Unique proteins in alignment graph
1. NetworkBLAST [15]						
*S. cerevisiae *	94.87	N/A	100	54	117	348
*D. melanogaster *	84.62	N/A	100	43	117	256
2. HopeMap-bestPairs						
*S. cerevisiae *	97.18	N/A	100	51	71	1314
*D. melanogaster *	76.06	N/A	100	34	71	1314
3. HopeMap—data from 1						
*S. cerevisiae *	98.73	N/A	100	54	79	1645
*D. melanogaster *	78.48	N/A	100	39	79	1913
4. HopeMap-KO						
*S. cerevisiae *	100.00	100	N/A	20	26	747
*D. melanogaster *	92.31	100	N/A	19	26	753

**Table 4:** Specificity and sensitivity results for *eco/stm* and *eco/ccr* from HopeMap.

Species	Specificity (%) in GO	Specificity in KO (%)	Number of GO categories enriched (*P* value < 0.05)	Total conserved regions	Number of unique proteins in alignment graph
*E. coli* K12 (eco)	100	100	49	58	2085
*S. typhimurium* (stm)	96.55	100	46	58	2183
*E. coli* K12 (eco)	95.24	100	37	42	1069
*C. crescenttus* (ccr)	90.48	100	31	42	1138

**Table 5:** Specificity comparison in terms of KO groups for different alignment tools.

Tools	*eco/stm *		*eco/cce *		*sce/dme *	
	*C*_eq_	*C*_node_	*C*_eq_	*C*_node_	*C*_eq_	*C*_node_
GrG	0.86	0.86	0.72	0.72	0.68	0.68
ISO	0.91	0.91	0.65	0.65	0.63	0.63
Gr2.0	0.96	0.96	0.78	0.78	0.73	0.73
HopeMap	1.0	1.0	1.0	1.0	1.0	1.0

**Table 6:** Sensitivity comparison in terms of KO groups.

Tools	*eco/stm *		*eco/cce *		*sce/dme *	
	*C*_or_	Tot	*C*_or_	Tot	*C*_or_	Tot
GrG	1496		720		384	
ISO	2026		1014		534	
Gr2.0	2024		1012		637	
HopeMap	2159	3151	1061	1365	768	1664

**Table 7:** A comparison of networkBLAST, NetworkBLAST-M, and HopeMap for *yeast/fly/worm*.

Species	Specificity (%) in GO	Number of GO categories enriched (*P* value < 0.05)	Total conserved regions
NetworkBLAST			
*S. cerevisiae *	100	14	59
*C. elegans *	88	13	59
*D. melanogaster *	94.9	16	59
NetBLast-M restriceted			
*S. cerevisiae *	100	29	64
*C. elegans *	68.8	32	64
*D. melanogaster *	98.4	37	64
NetBLASt-M relaxed order			
*S. cerevisiae *	94.6	45	92
*C. elegans *	67	29	92
*D. melanogaster *	90.1	41	92
HopeMap-KO			
*S. cerevisiae *	100	15	18
*C. elegans *	80	5	18
*D. melanogaster *	88.89	14	18

**Table 8:** Specificity in MIPS for homologues cluster of *yeast/fly* in HopeMap.

Type		Note
Homolog clusters	71	Results of HopeMap-bestPairs in Table 3 for yeast and fly
MIPS complexes	70%	percent of clusters in the same complexes of MIPS for yeast (not available for fly)
MIPS FunCats	86%	Percent of clusters in the same FunCats, using 28 main levels of FunCats in MIPS for Yeast
MIPS FunCats	76%	Percent of clusters in the same FunCats using 28 main levels of FunCats in MIPS for fly and GO2MIPS conversion (excluding 30 proteins in fly without available FunCats)

**Table 9:** The run-time results for pairwise alignment.

Number of species	Number of proteins	Number of PPI edges	Number of nodes	Runtime (sec)
2	8360	311035	3151	35.21
